# Different immunological patterns of Down syndrome patients with and without recurrent infections

**DOI:** 10.1016/j.jped.2024.06.007

**Published:** 2024-07-22

**Authors:** Kamila Rosa Martins, Flavia Araujo Alves, Luiz Roberto da Silva, Lauren Olivia Alves da Silva, Gesmar Rodrigues Silva Segundo

**Affiliations:** a Universidade Federal de Uberlândia, Hospital de Clínicas, Uberlândia, MG, Brazil; b Universidade Federal de Uberlândia, Uberlândia, MG, Brazil; c Universidade Federal de Uberlândia, Faculdade de Medicina, Departamento de Pediatria, Uberlândia, MG, Brazil

**Keywords:** Downʼs syndrome, Recurrent infections, Immunology, Pneumococcal vaccines

## Abstract

**Objective:**

Individuals with Down Syndrome (DS) exhibit a higher susceptibility to infections, suggesting potential immunological alterations within this population. Consequently, this study aims to assess the immune response profile in children with DS to identify potential immune dysfunctions associated with recurrent infections.

**Methods:**

The authors conducted a retrospective analysis involving 49 DS patients, examining various epidemiological, clinical, cytogenetic, and laboratory variables. The studyʼs sample comprised patients aged 2–20 years, with a predominance of males. These patients were categorized into two groups based on the presence or absence of recurrent infections, as indicated by the Jeffrey Modell Foundation alert signs.

**Results:**

Immunoglobulin (Ig) A, G, and M levels were deemed normal, although individuals with DS experiencing recurrent infections exhibited significantly lower IgA levels. Additionally, CD3, CD4, CD8, and CD19 lymphocyte counts were found to be within normal ranges, with no significant differences between the two groups. While overall data indicated normal seroconversion levels of pneumococcal polysaccharide antibodies, a notable impairment in seroconversion was observed among DS patients with recurrent infections compared to those without such infections.

**Conclusion:**

The deficiency of anti-polysaccharide antibodies in individuals with DS may constitute an important immunological comorbidity. Therefore, it warrants further investigation, particularly among individuals with recurrent infections.

## Introduction

Down Syndrome (DS), also known as trisomy 21, is reported as one of the most frequent autosomal chromosomal abnormalities, with easily recognizable phenotypic particularities.^1^ According to the Brazilian Institute of Geography and Statistics, DS affects 5.8 million people worldwide^2^ and approximately 300,000 in Brazil. The syndrome incidence is 1 for every 660 live births and occurs without distinction of sex, ethnicity, or socioeconomic class.^3^ DS is also considered the most common genetic cause of intellectual disability with several specific health conditions.^4^

Respiratory tract infection diseases, including pneumonia caused mainly by *Streptococcus pneumoniae*, are the main cause of morbidity and mortality in children with DS.^5^ In DS infants, respiratory infections are the main cause of recurrent hospitalization. Around 88 % of patients experienced hospitalization due to respiratory infections and about 16 % presented with recurrent hospitalizations including intensive care requirements.^6^

The immune system in DS patients is considered disorganized. Changes in the number of immune cells, including T and B cells, monocytes, neutrophils, and lower vaccine response were previously described, however, the mechanisms leading DS patients to present with respiratory and other infections and frequent hospitalizations have not been fully understood.^6^

Thus, this study aimed to evaluate the immune response profile of children with DS with and without recurrent infection, trying to recognize possible immune alteration associated with the presence of recurrence infection.

## Methods

An observational, cross-sectional, and retrospective study was carried out using the medical records of patients with DS evaluated at the Down Syndrome Outpatient Clinic of a University Hospital, from 2016 to 2019. The study was approved by the Research Ethics Committee of the Federal University of Uberlândia (CAAE: 92851018.0.00005152).

Sociodemographic data, clinical variables, perinatal history, cytogenetics, information on comorbidities, history of infections, vaccination history, and results of laboratory tests described were collected from the medical records. As a routine, DS patients in the studied institution usually are investigated for immunological alterations including immunoglobulins, lymphocyte subpopulations, and vaccine antibody response.

All patients with DS, over 2 years old, attended the DS outpatient clinic from 2016 to 2019, with complete immunological screening, and participated in the research. Children under 2 years of age, incomplete medical records, and absence of immunological assessment data were excluded from the study. Patients were separated into two groups: with and without recurrent infection according to the 10 warning signs from the Jeffrey Modell Foundation, as well as its adaptation for Brazil^7^ such as two or more pneumonia in the year; four or more ear infections in the last year; two or more serious sinusitis per year, need for intravenous antibiotics for infections, recurrent stomatitis, or moniliasis for more than two months; recurrent abscesses; an episode of severe systemic infection (meningitis, osteoarthritis, septicemia); recurrent intestinal infections/chronic diarrhea and severe asthma.

Immunological lab data: lymphocyte subpopulations (CD3+, CD4+, CD8+, CD19+) and immunoglobulins (IgA, IgM, and IgG). The authors also analyzed the results of antibodies against pneumococcal polysaccharide vaccine (serotypes 4, 6B, 9V, 14, 18C, 19F, 23F) pre-vaccination and 6 weeks after the application of the 23-valent pneumococcal capsular polysaccharide vaccine, considered as post-vaccination. An adequate response to polysaccharide was defined as a post-immunization antibody concentration equal to or greater than 1.3 µg/mL or an increase of at least 4-fold compared to baseline.^8^^,^^9^

### Statistical analysis

Qualitative categorical variables were analyzed using *Fisherʼs exact test* and expressed as percentages. To test the normality of the distributions, the *Kolmogorov–Smirnov test* was used. Quantitative numerical variables with non-normal distribution were described as median and confidence interval. The *Mann–Whitney* and *Fisherʼs Exact tests* were used, with a significance level of 5 % (*p* < 0.05). Analyzes were performed using the software *GraphPad Prism* 9.0.1 (2021).

## Results

From 64 patients followed at the Down Syndrome Outpatient Clinic of the HC-UFU from 2016 to 2019, 49 DS patients aged 2–20 years were enrolled. Fifteen patients were excluded due to the lack of immunological laboratory data in their medical records. In the final group, 29 (59.2 %) were male, simple trisomy was the most commonly found (91.8 %), followed by three cases of translocation (6.1 %), and only one case of mosaicism (2.1 %) in the cytogenetic study (Table 1). The maternal age group between 36 and 40 years was the most frequent (34.7 %), most births were at term (67.3 %) and the most used mode of delivery was cesarean (79.6 %). Regarding gestational complications, 21 pregnant women had some type of event, with urinary tract infections being more frequent. Suspicion of DS was evidenced in the postnatal period in 45 (91.8 %) births. In the analysis of the parents' degree of kinship, only 1 couple had consanguinity (Table 1).

**Table 1:** Gender, karyotype, gestational, and birth data of patients with Down Syndrome seen at the Down Syndrome Outpatient Clinic of HC-UFU from 2016 to 2019.

	Frequency		
		n	%
Gender	Female	20	40.8
	Male	29	59.2
Karyotype	Free Trisomy	45	91.8
	Translocation	3	6.1
	Mosaicism	1	2.1
Maternal age during gestation (years)	< 25	6	12.2
	25–30	11	22.5
	31–35	6	12.2
	36–40	17	34.7
	41–45	9	18.4
Gestational time of delivery	Preterm	14	28.6
	Full term	33	67.3
	Post-term	2	4.1
Syndrome confirmation	Prenatal	4	8.2
	Post-natal	45	91.8
Type of birth	Cesarean Section	39	79.6
	Vaginal	10	20.4

n: absolute number;%: percentage.

Thirty-one (63.3 %) children had perinatal complications, including jaundice (64.5 %) and respiratory distress (22.5 %). The distribution of weight and height percentiles of DS patients, according to Bertapelli^10^ curves showed birth weight above 2.5 kg in 67.3 % of patients, with 31 % of male children weighing between the 25th and 50th percentiles and 35 % of female children were between the 50th and 75th percentiles of the Down Syndrome chart for the Brazilian population. As for height, 31 % of males were between the 10th and 25th percentiles, while 45 % of females were between the 25th and 50th. Immunological labs showed mean IgG (1094 mg/dl), IgA (150 mg/dl), and IgM (66 mg/dl), all considered normal for specific ages according to the Brazilian infantile immunoglobulins chart.^11^

After the initial analysis, patients were divided into two groups: 25 patients with recurrent infection (Group 1- Warning signs data are shown in Table 1S) and 24 patients without a history of recurrent infection (Group 2). Levels of IgG and IgM levels were considered normal for the specific ages, group 1 and group 2 did not show significant differences between their values. On the other hand, serum IgA levels were also considered normal for specific ages in each group, however, group 1 (median 133 mg/dL, IQR = 71 – 173) were significantly lower than the serum levels of DS children without infection (median 149.5 mg/dL, IQR = 119–183) with *p* = 0.0020. Another interesting finding, no DS patients showed important IgE elevation associated with aeroallergen sensitization or food sensitization. The data on CD3, CD4, and CD8 lymphocytes showed values also considered normal for age, while CD19 lymphocytes were slightly reduced compared with normal children at the same ages^12^ and no difference was found between the groups 1 and 2 (Table 2).

**Table 2:** Median and confidence interval of immunoglobulin, lymphocytes, and seroconversion vaccine percentage values of patients with DS with (group I) and without (group II) recurrent infection seen at the Down Syndrome Outpatient Clinic of HC-UFU from 2016 to 2019.

	Group I (*n* = 25)		Group II (*n* = 24)		
	Median	IC	Median	IC	*p-value*
Age (months)	63	45–111	86	69–113	0.0773
Serum Immunoglobulins					
IgG	1027	885–1256	1032	933–1130	0.6880
IgA	133	71–173	149.50	119–183	0.0020*
IgM	63	40–82	61.75	48–74	0.8809
IgE total	10	1.84–63	61	7–531	0.2212
Lymphocytes					
CD3	1838	1247–2156	1427	1240–1992	0.4379
CD4	811.5	547–974	731	559–845	0.1780
CD8	1001	579–1294	571	440–1106	0.3945
CD19	174	117–214	162.5	123–238	0.5379
Post-pneumococcal polysaccharide vaccine seroconversion (%)	28.6	14.3–57.2	92.9	42.9–100	0.0086*

Mann–Whitney test; IC: interval confidence; * *p*-value < 0.05.

**Figure 1:**
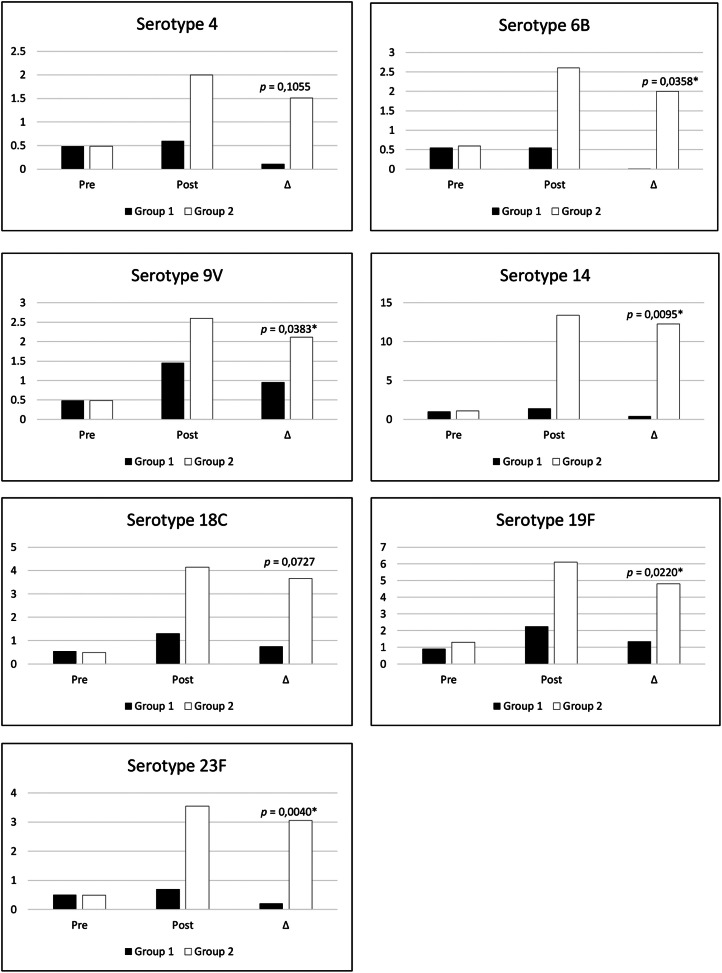
Levels of specific IgG (mg/dl) for pneumococcal serotypes before and after pneumo23 vaccine in DS patients with recurrent infections (group 1, black bars) and without recurrent infections (group 2, white bars). Statistical differences among pos vaccine levels were found for serotypes 6B (*p* = 0.0278), 14 (*p* = 0.0022), 19F (*p* = 0.0338), and 23F (*p* = 0.0025). Δ indicates the variation levels in the specific IgG before and after vaccines and showed statistical differences for serotypes 6B, 9V, 14, 19F, and 23F (*p < 0.05).

Post pneumococcal polysaccharide vaccine antibodies evaluation showed a median frequency of seroconversion in 28.60 % (14.3 – 57.2) of serotypes evaluated in group 1 (recurrent infections) and 92.90 % (42.9–100.0) in group 2 (no recurrent infections), showing significant statistic difference (*p* = 0.0086) between groups. The analyses of each specific serotype response showed significant differences in the post-vaccine levels of serotypes 6B (*p* = 0.0278), 14 (*p* = 0.0022), 19F (*p* = 0.0338), and 23F (*p* = 0.0025). The authors also verified significant differences for the pre and post-variation of IgG levels (delta) for serotypes 6B, 9 V,14,19, and 23 as shown in Figure 1.

Pathologies associated with DS were found in most patients and showed no significant differences between groups (Table 3). Forty-three (87.7 %) patients had changes in the echocardiogram, and 25 had undergone surgical intervention for some type of cardiac correction. Endocrine alterations were seen in 37 (75.5 %) patients, with hypothyroidism being the main pathology. Refraction impairment was more frequent in ophthalmic alterations and affected 21 (42.8 %) patients. Lymphopenia and benign childhood neutropenia were the main findings in the analysis of the hematological system.

**Table 3:** Distribution of the main comorbidities and cardiac surgery of patients with DS with (group I) and without (group II) recurrent infection treated at the Pediatric Outpatient Clinic of HC-UFU from 2016 to 2019.

	Group II (*n* = 24)		Group I (*n* = 25)		OR	IC	*P- value*
	n	%	n	%			
**Cardiovascular System**	22	(88.0 %)	21	(87.5 %)	1.048	0.2244–4.881	>0.9999
**Cardiologic Surgery Intervention**	15	(68.2 %)	10	(47.6 %)	2.357	0.7290–8.776	0.2231
**Endocrine System**	21	(84.0 %)	16	(66.7 %)	2.625	0.6778–8.779	0.1963
**Ophthalmic abnormalities**	9	(36.0 %)	12	(50.0 %)	0.5625	0.1956–1.810	0.3931
**Hematological System**	8	(32.0 %)	5	(20.8 %)	1.788	0.5035–6.156	0.5202
**Nervous System**	6	(24.0 %)	3	(12.5 %)	2.211	0.5521–8.813	0.4635
**Genital System**	2	(8.0 %)	4	(16.7 %)	0.4348	0.07769–2.067	0.4174
**Locomotor System**	2	(6.7 %)	4	(16.7 %)	0.4348	0.07769–2.067	0.4174
**Abdominal Wall Changes**	1	(4.0 %)	3	(12.5 %)	0.2917	0.02169–2.132	0.3487
**Hearing Aid**	2	(8.0 %)	3	(12.5 %)	0.6087	0.1014–3.250	0.6671
**Digestive System**	3	(12.0 %)	1	(4.17 %)	3.136	0.4299–42.26	0.6092
**Urinary System**	0		0		—	—	>0.9999

Fischerʼs exact test; OR: odds ratio; IC: interval confidence; * *p*-value < 0.05.

## Discussion

The understanding of the reasons why patients with DS have a greater number of respiratory infections than other children has been the subject of several researchers. In the follow-up of patients with DS, the responsible physicians notice a difference among patients with the syndrome, as well as in other children, with a group of them having recurrent infections, while another group has a frequency and severity of infections similar to children without the syndrome. For this reason, the authors decided to study patients with DS comparing them to other patients with DS, reporting them in groups with and without recurrent infections, using the warning signs for primary immunodeficiencies.

General data about gender with a male predominance and cytogenetic showing simple/free trisomy above 90 % of DS cases is according to prior published data.^13^^,^^14^ Previous studies showed an increased incidence of births with DS related to advancing maternal age and demonstrated an increased risk of 6.5 times between 35 and 39 years when compared to the group aged 20–24 years, and 20.5 times in the range between 40 and 44 years old.^15^ The present study found similar data showing more than 53.1 % of patients born from mothers over 35 years old. Also, similar to the literature, the DS suspicion was raised in most births in the postnatal period (91.8 %).^16^ The rate of C-sections reported here is considered high compared to other countries and occurs across all populations in Brazil without a relationship to the DS patients.^17^

Patients with DS have comorbidities with high frequency, especially cardiologic and endocrinologic, and require screening for several diseases.^18^^,^^19^ In the present study, 87.7 % of DS patients presented echocardiogram alterations and 51.0 % had undergone surgical intervention for some type of cardiac correction. These findings showed an elevated percentage of cardiac diseases in comparison with earlier data that showed 40–50 % of this population manifest some type of heart disease at birth.^19^ Some previous studies demonstrated the association between congenital heart diseases and recurrent infections in DS patients.^20^^,^^21^ This data, different from previous papers, did not show the difference in the presence of cardiac alterations or cardiac surgery intervention between DS groups with or without recurrent infections. Other comorbidities also did not demonstrate differences between DS groups.

The increased susceptibility to infections in DS, especially respiratory, has been reported in several studies, and immunological changes that might explain the frequency and severity of infections in this group have been studied for some time.^22^^,^^23^ The present study found normal levels of serum immunoglobulins (IgG, IgM, and IgA) for specific ages. A few studies showed an IgM level decreased, especially during adolescence, compared with the general population, while the present study verified the median levels of IgM in the tenth percentile considering the median age of patients.^24^^,^^25^ Some publications demonstrated an increased IgG level in children with DS, while this study showed median levels of IgG around the percentile 75, again, considering the median age of patients.^5^^,^^24^^,^^25^ Kusters et al. initially found normal values and increased them after 6 years of age in the population with DS.^26^

The present results also showed normal values for CD3, CD4, and CD8, and a slight reduction in CD19, which is in agreement with other studies that express a decrease in CD19 B lymphocytes in the DS population.^25^ Studies reveal that the total number of circulating T lymphocytes is normal or slightly decreased in syndromic individuals and shows variations in some subpopulations when compared to a control group of the same age and without chromosomal alterations.^24^^,^^27^^,^^28^ Around 10 years of age, T lymphocytes tend to reach the values of normal controls, whereas B lymphocytes remain below the normal values of control children at all ages.^26^

To our knowledge, this is the first study comparing groups of patients with DS with and without recurrent infections. Although the authors did not observe a difference in other immunological analyses, the response to pneumococcal polysaccharide vaccine showed a reduced significant difference between DS groups with infections compared with those without infections. Other studies reported a lower seroconversion in the DS population but considered their levels as normal, different from the findings shown here.^22^^,^^29^ A recent study also showed reduced levels of pneumococcal antibodies levels in DS patients with recurrent infections and one or more of the 10 warning signs from the Jeffrey Modell Foundation, however, they did not perform a response to pneumococcal polysaccharide vaccine with pre- and pos-vaccine antibodies dosages, just an isolated antibody levels measurement.^14^ The probable cause of normal levels of pneumococcal response in the previous studies occurred due to the participation of patients with DS with and without infections, and the higher levels of seroconversion in the group without infections raises the average and places it within the limits of normality.

The inability to produce polysaccharide antibodies after receiving the pneumococcal polysaccharide vaccine characterizes the anti-polysaccharide antibody deficiency, one of the most common primary immunodeficiencies in childhood, and the present data suggests a higher frequency in DS patients.^30^

One important finding of the present study is regarding the type of pneumococcal serotypes analyses. All seven serotypes analyzed are presented in the conjugate vaccines and, even though all of the patients received conjugate vaccines in the first and second year of life, the group with infections was not able to show a booster after polysaccharide pneumococcal vaccine, showing these serotypes could be used to evaluate the response in patients previously vaccinated with pneumococcal conjugate vaccines.

The present study has limitations as to being done in a single center, the number of participants, and the retrospective format are also limiting points, however, with minor impact on the final findings. Another possible limitation could be the post-pneumococcus polysaccharide vaccination response analysis of only 7 serotypes, especially those included in conjugate vaccines. However, the poor response after the pneumococcal polysaccharide vaccine to these serotypes further reinforces the presence of alterations in the antibody response in a group of patients with DS.

In conclusion, patients with DS present variability of comorbidities, and the deficiency of anti-polysaccharide antibodies post-immunization seems to be an important immunological comorbidity and should be investigated in DS patients with recurrent infections. The 10 warning signs for primary immunodeficiency from the Jeffrey Modell Foundation could be used to guide physicians about patients needing investigation. Although not the purpose of the study, DS patients with recurrent infections and deficiency of anti-polysaccharide antibodies presented here have been treated with prophylactic antibiotic therapy and/or immunoglobulin replacement as previously described,^30^ with significant control of infections and improvement in quality of life, demonstrating the importance of appropriate medical intervention.

## Funding

Jeffrey Modell Foundation - CHILDREN Program.

## Declaration of generative AI and AI-assisted technologies in the writing process

During the preparation of this work, the author(s) used Grammarly I.A. in order to improve language and readability. After using this tool/service, the authors reviewed and edited the content as needed and take full responsibility for the content of the publication.

## Conflicts of interest

The authors declare no conflicts of interest.

## Appendix Supplementary materials

Image, application 1

## Footnotes

Supplementary material associated with this article can be found in the online version at doi:10.1016/j.jped.2024.06.007.
