# Supplementary material for: Different immunological patterns of Down syndrome patients with and without recurrent infections
Source: J Pediatr (Rio J). 2024 Jul 22;100(6):653–9. doi: 10.1016/j.jped.2024.06.007 (PMC11662752; doi:10.1016/j.jped.2024.06.007)
Supplement: Supplementary file 1 [file mmc1.docx]

**JPED-D-23-00512 – Supplementary Material**

**Table Suplementary 1** Down Syndrome patients with recurrent infections and one or more warning signs for Primary Immunodeficiencies.

| JMF Warning Sign | Patients n (%) |
| --- | --- |
| > 4 ear infections in one year | 9 (36%) |
| > 2 severe sinus infections in one year | 9 (36%) |
| > 2 months of antibiotics with little effect | 6 (24%) |
| > 2 pneumonias per year | 14 (56%) |
| Need for intravenous antibiotics to clear infections | 12 (48%) |
| > 2 deep-seated infections | 3 (12%) |
| Recurrent deep skin or organ abscess* | 6 (24%) |
| Insufficient weight gain or growth delay | 0 (0%) |
| Persistent thrush in mouth or fungal infection on skin | 0 (0%) |
| Family history of a primary immunodeficiency | 0 (0%_ |
| Brazilian modifications |  |
| An episode of severe systemic infection (meningitis, osteoarthritis, septicemia) | 3 (12%) |
| Intestinal infections/chronic diarrhea | 1 (4%) |
| Severe asthma or auto-immune disease | 0 (0%) |
| BCG adverse effect or Mycobacteria infection | 0 (0%) |
| recurrent stomatitis, or moniliasis for more than two months | 0 (0%) |

* Only deep skin abscesso.
